# Intravenous infusion route in maternal resuscitation: a scoping review

**DOI:** 10.1186/s12873-021-00546-9

**Published:** 2021-12-03

**Authors:** Eishin Nakamura, Shinji Takahashi, Shigetaka Matsunaga, Hiroaki Tanaka, Marie Furuta, Atsushi Sakurai, the Japan Resuscitation Council (JRC), Guideline Editorial Committee

**Affiliations:** 1 Center for Maternal, Fetal and Neonatal Medicine, Saitama Medical Center, Saitama Medical University, 1981 Kamoda, Kawagoe-shi, Saitama, 350-8550 Japan; 2 Japan Resuscitation Council, Maternal group, Tokyo Japan, 2-5-4 Yoyogi, Sibuya-ku, Tokyo, 151-0053 Japan; 3 Department of Anesthesiology, Juntendo University Urayasu Hospital, 2-1-1 Tomioka, Urayasu-shi, Chiba, 279-0021 Japan; 4 Department of Obstetrics and Gynecology, Saitama Medical Center, Saitama Medical University, 1981 Kamoda, Kawagoe-shi, Saitama, 350-8550 Japan; 5 Department of Obstetrics and Gynecology, Mie University School of Medicine, 2-174 Edobashi, Tsu-shi, Mie 514-8507 Japan; 6 Department of Human Health Sciences, Graduate School of Medicine, Kyoto University, 53 Kawahara-cho Shogo-in, Sakyo-ku, Kyoto, 606-8507 Japan; 7 Division of Emergency and Critical Care Medicine, Department of Acute Medicine, Nihon University School of Medicine, 30-1 Oyaguchi Kamimachi, Itabashi-ku, Tokyo, 173-8610 Japan

**Keywords:** Cardiopulmonary resuscitation, Pregnancy, Resuscitation, Intravenous infusion, Scoping review

## Abstract

**Background:**

The concept that upper extremities can be used as an infusion route during cardiopulmonary resuscitation in pregnant women is a reasonable recommendation considering the characteristic circulation of pregnant women; however, this method is not based on scientific evidence.

**Objective of the review:**

We conducted a scoping review to determine whether the infusion route should be established above the diaphragm during cardiopulmonary resuscitation in a pregnant woman.

**Discussion:**

We included randomized controlled trials (RCTs) and non-RCTs on the infusion of fluids in pregnant women after 20 weeks of gestation requiring establishment of an infusion route due to cardiac arrest, massive bleeding, intra-abdominal bleeding, cesarean section, severe infection, or thrombosis. In total, 3150 articles from electronic database were extracted, respectively. After title and abstract review, 265 articles were extracted, and 116 articles were extracted by full-text screening, which were included in the final analysis. The 116 articles included 78 studies on infusion for pregnant women. The location of the intravenous infusion route could be confirmed in only 17 studies, all of which used the upper extremity to secure the venous route.

**Conclusion:**

Pregnant women undergo significant physiological changes that differ from those of normal adults, because of pressure and drainage of the inferior vena cava and pelvic veins by the enlarged uterus. Therefore, despite a lack of evidence, it seems logical to secure the infusion route above the diaphragm when resuscitating a pregnant woman.

**Supplementary Information:**

The online version contains supplementary material available at 10.1186/s12873-021-00546-9.

## Background

The Japan Resuscitation Council (JRC) has published resuscitation guidelines in 2010, 2015, and 2020. ITherefore, a new algorithm for resuscitation of pregnant women was developed. Although the number of maternal deaths in Japan is approximately 4 per 100,000 deliveries [1], the effect of circuatory dynamics of pregnant women generates a need to determine effective and evidence-based maternal resuscitation methods.

The American Heart Association (AHA) developed and illustrated an algorithm based on the International Consensus Conference on Cardiopulmonary Resuscitation and Emergency Cardiovascular Care Science With Treatment Recommendations (CoSTR) [2], which stated that intravenous access should be considered above the diaphragm [3]. This statement is justifiable considering the special circulatory dynamics of pregnancy, but we believe that it needs to be supported by evidence and not by a scientific recommendation based on evidence.

However, there are no validated randomized controlled trials (RCTs) on the choice of infusion route during maternal resuscitation, and most studies have not included cardiac arrest cases. Pregnant women have a particular circulatory situation in which the inferior vena cava is compressed by the enlarged uterus, so the usual choice of infusion route during resuscitation of adults may not be applicable.

Therefore, we decided to conduct a comprehensive review of the practicality of the infusion route in pregnant women with the purpose of scientifically supporting whether the infusion route should be secured above the diaphragm during pregnancy. We created a clinical question (CQ) from the algorithm and 2015 statements of the American Heart Association (AHA). This report is a scoping review of the question, “In cardiopulmonary resuscitation of pregnant women, should the infusion route be taken above the diaphragm for massive infusion?”

## Methods

The maternal group within the JRC of the Guideline Editorial Committee established the CQ. However, since there are no valid studies on the location of the infusion route during resuscitation of pregnant women, we concluded that a systematic review would be difficult and decided to instead conduct a scoping review of all studies on the infusion route for pregnant women.

### Protocol and registration

This study was systematized and conducted concerning the Preferred Reporting Items for Systematic reviews and Meta-Analyses (PRISMA) extension for Scoping Reviews checklist [4].

### Eligibility criteria

Only articles published in peer-reviewed journals were included in the review, and articles consisting only of abstracts were excluded. Only literature published in English were included, while articles published in other languages were excluded. Literature on animals were excluded, and only studies on humans were included. The types of studies included were RCTs, non-RCTs (such as split time-series analyses, before-and-after comparative studies, cohort studies, case reports, and meta-analyses), case-concentration studies, reviews, and existing guidelines. Unpublished studies (e.g., conference abstracts and clinical trial protocols) were excluded.

### Information sources

Articles published on or before December 07, 2019 were retrieved from MEDLINE/PubMed and Embase. The search strategy was constructed by Marie Furuta, PhD Health Studies, an expert in public health (co-author of this paper).

### Search

The search strategy is shown in Supplementary Material 1.

### Selection of sources of evidence

Data charting of each literature was performed independently by two reviewers. In pairs sequentially evaluated the titles and abstracts, while screening of full-text articles were performed independently.

### Data charting process

Population, concept, and context frameworks were created [5], as shown in Table 1. A data charting form was jointly developed by two reviewers to determine the relevant articles that should be extracted.

**Table 1:** PCC (Population, Concept, Context) frameworks

Population: A study on the infusion of fluids in pregnant women after 20 weeks of gestation.	
Concept: RCT and non-RCT (split time-series analysis, before-and-after comparative studies, cohort studies, case reports, and meta-analysis) were included in studies of patients requiring intravenous infusion or intraosseous infusion due to cardiac arrest, massive bleeding, intra-abdominal hemorrhage, cesarean section, severe infection, thrombosis.	
Context: We searched the literature, regardless of the region in which the study was conducted, race, or differences in health care systems by medical area.	

## Results

### Selection of sources of evidence

**Fig. 1:**
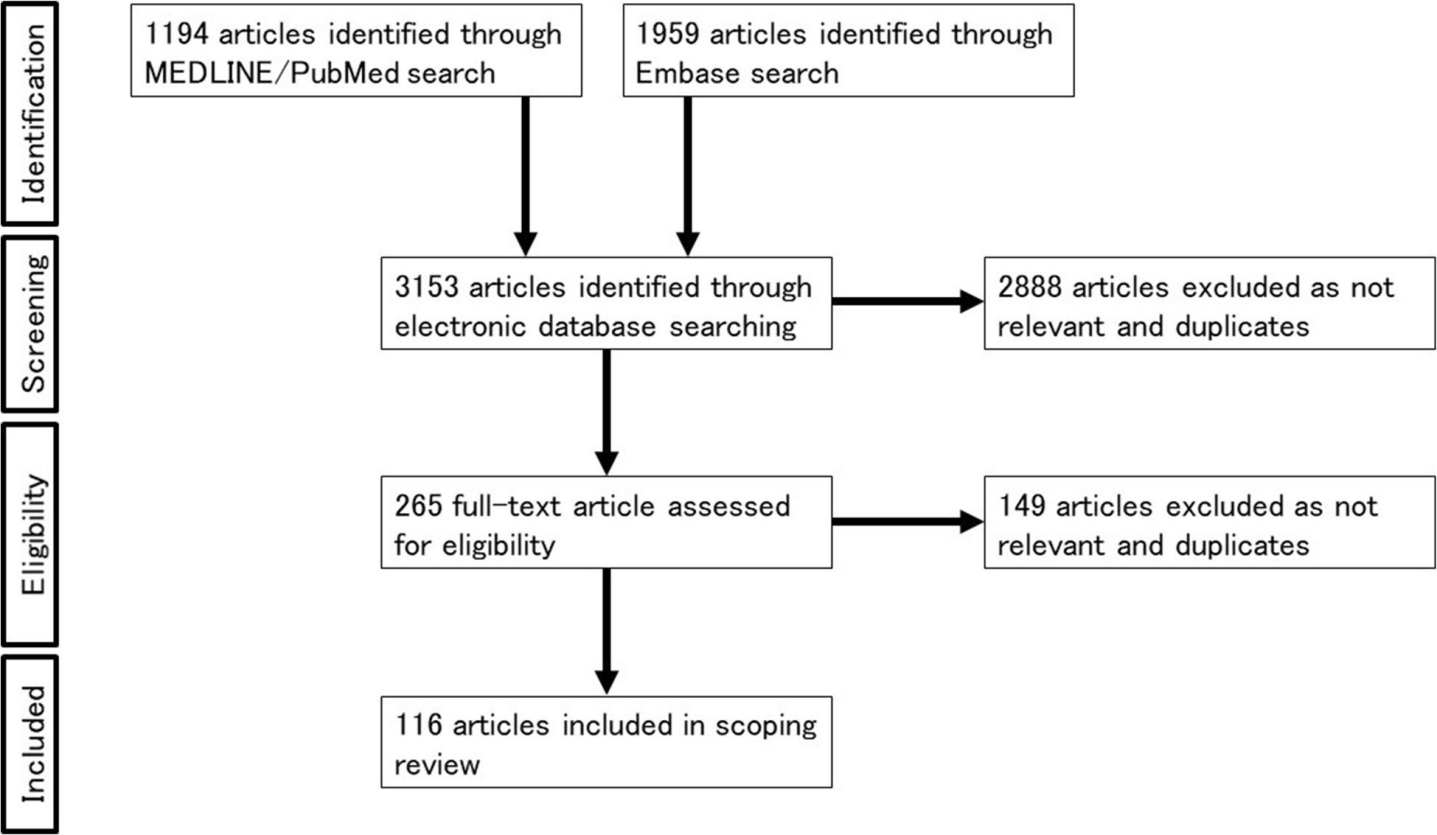
PRISMA flowchart of the study

Figure 1 shows the PRISMA flowchart [6] for this scoping review.

### Characteristics of sources evidence

The search for articles was performed on December 07, 2019 based on the search formula described in Supplementary Material 1. 1194 articles were extracted from MEDLINE/PubMed and 1959 articles were extracted from Embase. After title and abstract review, 265 articles met the eligibility criteria by deduplication and relevance screening. Supplementary Material 2 shows the list of references in which full-text screening was conducted. Full-text screening was then conducted, and 116 references were extracted for the final analysis.

### Results of individual sources of evidence

Table 2 presents the details of the breakdown of the 116 articles.

**Table 2:** General characteristics of included articles

		Types of literature (*n* = 116)			
		Caesarean section (*n* = 78)	Postpartum bleeding (*n* = 11)	Maternal cardiac arrest(*n* = 7)	Other complications in pregnancy†(*n* = 20)
Study design	RCT	54	5	0	6
	Prospective cohort study	15	0	0	2
	Retrospective cohort study	0	1	1	1
	Case control study	0	0	0	0
	Cross sectional study	0	0	0	3
	Case control study	0	0	0	0
	Case report and case series	0	0	4	0
	Meta analysis	3	0	0	0
	other	6	5	2	8

†Reports on the infusion route, including infections, thrombosis, and administration of pressure-boosting drugs in pregnant women

The 116 articles included 78 studies on the infusion of fluids during cesarean section, 11 on the infusion of fluids during hemorrhage, 7 on the infusion of fluids during cardiac arrest, and 20 on the infusion of fluids for infection, thrombosis, and other conditions in pregnant women based on the search formula described above. The location of the infusion route (upper or lower extremity) could be confirmed in only 17 studies, all of which used the upper extremity to secure the venous route. Table 3 shows a list of references that mention the infusion route.

**Table 3:** All references that mention the location of the infusion route

Author	Article type	Published year	Population	Location of the intravenous infusion route
Pouta et al. [13]	Randomized controled trial	1996	Pregnant woman undergoing a cesarean section	Upper extremities
King et al. [14]	Randomized controled trial	1998	Pregnant woman undergoing a cesarean section	Upper extremities
Ngan et al. [15]	Randomized controled trial	2000	Pregnant woman undergoing a cesarean section	Upper extremities
Desalu ei al [16].	Randomized controled trial	2005	Pregnant woman undergoing a cesarean section	Upper extremities
Ngan et al. [17]	Randomized controled trial	2005	Pregnant woman undergoing a cesarean section	Upper extremities
Ngan et al. [18]	Randomized controled trial	2008	Pregnant woman undergoing a cesarean section	Upper extremities
Nuthalapaty et al. [19]	Retrospective study	2009	Study on infusion for pregnant and postpartum patients	Upper extremities
Chatterjee et al. [20]	Case report	2011	Massive obstetric haemorrhage	Upper extremities
El-Mekawy et al. [21]	Randomized controled trial	2012	Pregnant woman undergoing a cesarean section	Upper extremities
Romdhani et al. [22]	Randomized controled trial	2014	Pregnant woman undergoing a cesarean section	Upper extremities
Cape et al. [23]	Retrospective study	2014	Patients with peripherally inserted central catheter during pregnancy	Upper extremities
Zasa et al. [24]	Randomized controled trial	2015	Pregnant woman undergoing a cesarean section	Upper extremities
Onwochei et al. [25]	Prospective study	2017	Pregnant woman undergoing a cesarean section	Upper extremities
Ngan et al. [26]	Randomized controled trial	2017	Pregnant woman undergoing a cesarean section	Upper extremities
Ngan et al. [27]	Randomized controled trial	2017	Pregnant woman undergoing a cesarean section	Upper extremities
Ngan et al. [28]	Randomized controled trial	2018	Pregnant woman undergoing a cesarean section	Upper extremities
Webster et al. [29]	Retrospective study	2018	massive obstetric hemorrhage	Upper extremities

We also conducted a comprehensive search for studies on maternal bone marrow tracts, but no relevant studies were identified.

### Summary of evidence

To conduct a broad review of maternal infusion routes, a scoping review was performed. Some literature, including RCTs, were found related to the infusion of fluids during surgery and bleeding in pregnant women. However, there were no RCTs related to maternal infusion routes, hence, a systematic review was decided against.

## Conclusions

In this study, we conducted a scpoing review to broadly review routes of maternal infusion. This comprehensive scoping review revealed that the number of studies mentioning the infusion route in detail is scarce, and the level of evidence for securing the infusion route above the diaphragm is small. However, there is no evidence of the adverse effects occurring when securing the infusion route above the diaphragm. The AHA recommendations further support an infusion route above the diaphragm, considering the pressure drainage of the inferior vena cava caused by an enlarged uterus in pregnant and nursing women, as detailed below.

The 2010 AHA Guideline, Part 12: Cardiac arrest in special situations, it was recommended that the infusion route be secured above the diaphragm [2].

The AHA likely came to this view considering the following special circulatory dynamics of pregnant and nursing women. Significant physiological changes occur in pregnant and nursing women, which differ from those in normal adults. In addition to increasing circulating blood volume up to 50%, decreasing peripheral vascular resistance, and increasing in cardiac output, the enlarged uterus causes pressure drainage of the inferior vena cava and pelvic veins, resulting in partial venous hypertension and edema [7].

It is justifiable to secure an infusion route above the diaphragm so that infusions and administered drugs can reach the heart without passing through the distended inferior vena cava [8].

Although these recommendations are reasonable based on the special circulatory dynamics of pregnant and nursing women, they are not based on evidence from the literature. Some studies have suggested that an infusion route below the diaphragm should be avoided because an enlarged uterus pressurizes and inhibits venous return [9].

It is noteworthy that the scoping review revealed that there are no valid RCTs on the choice of infusion route in pregnant women. A high-quality RCT of infusion routes during maternal resuscitation seems impossible due to the ethical difficulties of randomization and blinding. However, conducting RCTs and systematic reviews comparing upper and lower extremity infusion routes in the future, including in non-pregnant women, can be useful to build further evidence.

In non-pregnant women, there were three observational studies of adult out-of-hospital cardiac arrest with a total of 34,868 patients comparing the upper and lower extremity infusion routes [10–12]. Compared to the use of the venous route in the upper extremities, the use of the bone marrow route in the lower extremities was associated with worse outcomes such as poor survival, with < 17 patients discharged alive per 1000 cardiac arrest patients. These references compare the intravenous and intraosseous route with the target population being non-pregnant women. Although the above references need to consider inconsistency and indirection in this scoping review, it is possible that the outcome of cardiac arrest may be better if the infusion route is secured through the upper arm.

Pregnant women undergo significant physiological changes that differ from those of normal adults, due to pressure and drainage of the inferior vena cava and pelvic veins by the enlarged uterus. Therefore, despite a lack of evidence, it seems logical to secure the infusion route above the diaphragm when resuscitating a pregnant woman.

## Supplementary Information

**Additional file 1.** Supplementary Material 1: Detail of search strategy

**Additional file 2.** Supplementary Material 2: full-text reference list assessed for eligibility

## Data Availability

All data generated or analysed during this study are included in supplementary information files.

## Abbreviations

AHA: American Heart Association
CoSTR: International Consensus Conference on Cardiopulmonary Resuscitation and Emergency Cardiovascular Care Science With Treatment Recommendations
CQ: clinical question
PRISMA: Preferred Reporting Items for Systematic reviews and Meta-Analyses
RCTs: randomized controlled trials
