# Supplementary material for: Intravenous infusion route in maternal resuscitation: a scoping review
Source: BMC Emerg Med. 2021 Dec 3;21:151. doi: 10.1186/s12873-021-00546-9 (PMC8642880; doi:10.1186/s12873-021-00546-9)
Supplement: Supplementary file 1 — Additional file 1. Supplementary Material 1: Detail of search strategy [file 12873_2021_546_MOESM1_ESM.docx]

Database: Ovid MEDLINE(R) <1946 to December Week 1 2019>

--------------------------------------------------------------------------------

1 exp Pregnancy Complications, Cardiovascular/ or exp Pregnancy/ or exp Pregnancy, High-Risk/ or exp Pregnancy Complications/ (904441)

2 exp Pregnant Women/ (7775)

3 pregnan*.ab,ti. (441021)

4 matern*.ab,ti. (235378)

5 exp Maternal Mortality/ or exp Maternal Death/ (10387)

6 (maternal adj3 morbidit*).ab,ti. (6964)

7 exp Obstetrics/ (22200)

8 obstetric*.ab,ti. (82185)

9 Pregnant wom#n.ab,ti. (82348)

10 parturient.ab,ti. or exp Labor, Obstetric/ or exp Anesthesia, Obstetrical/ (58473)

11 peripartum.ab,ti. or exp Peripartum Period/ (4107)

12 exp Perinatology/ (1772)

13 Perinatal.ab,ti. (62300)

14 gestation*.ab,ti. (181138)

15 gravid*.ab,ti. (11863)

16 matern*.ab,ti. (235378)

17 1 or 2 or 3 or 4 or 5 or 6 or 7 or 8 or 9 or 10 or 11 or 12 or 13 or 14 or 15 or 16 (1123968)

18 exp Heart Arrest/ (46373)

19 (heart adj5 arrest*).ab,ti. (2335)

20 (cardiac adj5 arrest*).ab,ti. (28901)

21 (cardiopulmonary adj5 arrest*).ab,ti. (3933)

22 (cardiovascular adj5 arrest*).ab,ti. (309)

23 exp Cardiopulmonary Resuscitation/ (17226)

24 exp Resuscitation/ or exp Out-of-Hospital Cardiac Arrest/ (93256)

25 resuscita*.ab,ti. (53798)

26 (heart adj3 compression*).ab,ti. (238)

27 (cardiac adj3 compression*).ab,ti. (731)

28 (chest adj3 compression*).ab,ti. (3364)

29 exp Heart Massage/ (3087)

30 (heart adj3 massage*).ab,ti. (336)

31 (cardiac adj3 massage*).ab,ti. (1040)

32 (heart adj3 failure*).ab,ti. (140529)

33 (cardiac adj3 failur*).ab,ti. (16203)

34 (cardiovascular adj3 failur*).ab,ti. (1929)

35 (cardiopulmonary adj3 failure*).ab,ti. (743)

36 (cardiac adj3 collaps*).ab,ti. (234)

37 (cardiovascular adj3 collaps*).ab,ti. (1525)

38 (cardiopulmonary adj3 collaps*).ab,ti. (167)

39 cardiovascular.ab,ti. or exp Cardiovascular Diseases/ (2467011)

40 peri-arrest stat*.ab,ti. (1)

41 (life adj3 support*).ab,ti. (13760)

42 exp Emergencies/ or exp Emergency Medical Services/ (169658)

43 18 or 19 or 20 or 21 or 22 or 23 or 24 or 25 or 26 or 27 or 28 or 29 or 30 or 31 or 32 or 33 or 34 or 35 or 36 or 37 or 38 or 39 or 40 or 41 or 42 (2711828)

44 exp Administration, Intravenous/ or exp Infusions, Intravenous/ (141216)

45 (("intra?venous*" or venous) adj3 (route* or access* or insert* or device* or method* or fluid* or therap* or administer* or administrat* or infus* or drug* or medication*)).ab,ti. (136933)

46 exp Infusions, Intraosseous/ (706)

47 (("intra?osseous*" or venous) adj3 (route* or access* or insert* or device* or method* or fluid* or therap* or administer* or administrat* or infus* or drug* or medication*)).ab,ti. (13511)

48 44 or 45 or 46 or 47 (240601)

49 17 and 43 and 48 (1695)

50 limit 49 to (english language and humans) (1194)

***************************

Database: Embase <1996 to 2019 December 13>

Search Strategy:

--------------------------------------------------------------------------------

1 exp Pregnancy Complications, Cardiovascular/ or exp Pregnancy/ or exp Pregnancy, High-Risk/ or exp Pregnancy Complications/ (460698)

2 exp Pregnant Women/ (71465)

3 pregnan*.ab,ti. (475327)

4 matern*.ab,ti. (277410)

5 exp Maternal Mortality/ or exp Maternal Death/ (18753)

6 (maternal adj3 morbidit*).ab,ti. (11723)

7 exp Obstetrics/ (28808)

8 obstetric*.ab,ti. (106957)

9 Pregnant wom#n.ab,ti. (107459)

10 parturient.ab,ti. or exp Labor, Obstetric/ or exp Anesthesia, Obstetrical/ (31445)

11 peripartum.ab,ti. or exp Peripartum Period/ (36347)

12 exp Perinatology/ (1172)

13 Perinatal.ab,ti. (72035)

14 gestation*.ab,ti. (223811)

15 gravid*.ab,ti. (13477)

16 matern*.ab,ti. (277410)

17 1 or 2 or 3 or 4 or 5 or 6 or 7 or 8 or 9 or 10 or 11 or 12 or 13 or 14 or 15 or 16 (891278)

18 exp Heart Arrest/ (80403)

19 (heart adj5 arrest*).ab,ti. (2172)

20 (cardiac adj5 arrest*).ab,ti. (46220)

21 (cardiopulmonary adj5 arrest*).ab,ti. (5518)

22 (cardiovascular adj5 arrest*).ab,ti. (476)

23 exp Cardiopulmonary Resuscitation/ (92742)

24 exp Resuscitation/ or exp Out-of-Hospital Cardiac Arrest/ (95833)

25 resuscita*.ab,ti. (70861)

26 (heart adj3 compression*).ab,ti. (302)

27 (cardiac adj3 compression*).ab,ti. (855)

28 (chest adj3 compression*).ab,ti. (5887)

29 exp Heart Massage/ (1524)

30 (heart adj3 massage*).ab,ti. (93)

31 (cardiac adj3 massage*).ab,ti. (663)

32 (heart adj3 failure*).ab,ti. (239992)

33 (cardiac adj3 failur*).ab,ti. (20325)

34 (cardiovascular adj3 failur*).ab,ti. (3360)

35 (cardiopulmonary adj3 failure*).ab,ti. (1060)

36 (cardiac adj3 collaps*).ab,ti. (332)

37 (cardiovascular adj3 collaps*).ab,ti. (1795)

38 (cardiopulmonary adj3 collaps*).ab,ti. (225)

39 cardiovascular.ab,ti. or exp Cardiovascular Diseases/ (3318665)

40 peri-arrest stat*.ab,ti. (4)

41 (life adj3 support*).ab,ti. (19302)

42 exp Emergencies/ or exp Emergency Medical Services/ (121690)

43 18 or 19 or 20 or 21 or 22 or 23 or 24 or 25 or 26 or 27 or 28 or 29 or 30 or 31 or 32 or 33 or 34 or 35 or 36 or 37 or 38 or 39 or 40 or 41 or 42 (3476635)

44 exp intravenous drug administration/ (84227)

45 (("intra?venous*" or venous) adj3 (route* or access* or insert* or device* or method* or fluid* or therap* or administer* or administrat* or infus* or drug* or medication*)).ab,ti. (137888)

46 exp intraosseous drug administration/ (551)

47 (("intra?osseous*" or venous) adj3 (route* or access* or insert* or device* or method* or fluid* or therap* or administer* or administrat* or infus* or drug* or medication*)).ab,ti. (20990)

48 44 or 45 or 46 or 47 (202220)

49 17 and 43 and 48 (2438)

50 limit 49 to (human and english language) (1959)

--------------------------------------------------------------------------------

Supplemental data : Search strategy
